# Matrix Selection for the Visualization of Small Molecules and Lipids in Brain Tumors Using Untargeted MALDI-TOF Mass Spectrometry Imaging

**DOI:** 10.3390/metabo13111139

**Published:** 2023-11-09

**Authors:** Tianyao Lu, Lutz Freytag, Vinod K. Narayana, Zachery Moore, Shannon J. Oliver, Adam Valkovic, Brunda Nijagal, Amanda L. Peterson, David P. de Souza, Malcolm J. McConville, James R. Whittle, Sarah A. Best, Saskia Freytag

**Affiliations:** 1 Personalised Oncology Division, The Walter and Eliza Hall Institute of Medical Research, Melbourne 3052, Australia; 2 Department of Medical Biology, University of Melbourne, Melbourne 3052, Australia; 3 Metabolomics Australia, Bio21 Molecular Science and Biotechnology Institute, The University of Melbourne, Melbourne 3010, Australia; 4 Department of Biochemistry and Molecular Biology, Bio21 Institute of Molecular Science and Biotechnology, University of Melbourne, Melbourne 3010, Australia; 5 Department of Medical Oncology, Peter MacCallum Cancer Centre, Melbourne 3052, Australia

**Keywords:** MALDI-TOF, atmospheric pressure, matrix selection, brain cancer, glioma

## Abstract

Matrix-assisted laser desorption/ionization mass spectrometry imaging allows for the study of metabolic activity in the tumor microenvironment of brain cancers. The detectable metabolites within these tumors are contingent upon the choice of matrix, deposition technique, and polarity setting. In this study, we compared the performance of three different matrices, two deposition techniques, and the use of positive and negative polarity in two different brain cancer types and across two species. Optimal combinations were confirmed by a comparative analysis of lipid and small-molecule abundance by using liquid chromatography–mass spectrometry and RNA sequencing to assess differential metabolites and enzymes between normal and tumor regions. Our findings indicate that in the tumor-bearing brain, the recrystallized α-cyano-4-hydroxycinnamic acid matrix with positive polarity offered superior performance for both detected metabolites and consistency with other techniques. Beyond these implications for brain cancer, our work establishes a workflow to identify optimal matrices for spatial metabolomics studies.

## 1. Introduction

Brain cancer is a devastating malignancy that is currently incurable, with significant adverse effects on the patient’s quality of life. Like many other cancers, tumor cells in brain cancer display an altered metabolism, with an increase in the tricarboxylic acid (TCA) cycle related to tumor progression [1]. One of the metabolic pathways that is commonly altered is the switch to aerobic glycolysis, also known as the Warburg effect [2]. This metabolic adaptation, which is typically observed in healthy proliferating cancer cells, allows the cells to produce energy more efficiently, resulting in rapid cell growth and division. Other metabolic alterations include the production of the onco-metabolite 2-hydroxyglutarate (2-HG) as a result of a point mutation in the isocitrate dehydrogenase 1/2 (*IDH1/2*) gene, strongly associated with a discrete subset of brain cancers (*IDH*-mutant gliomas) [3,4,5]. 2-HG inhibits the activity of enzymes that are involved in DNA and histone modifications, leading to epigenetic changes in gene expression that promote cancer cell growth and survival.

Metabolic activity is not uniformly altered in brain cancer tumors, reflecting complex and multifactorial regulation that in turn depends on the cellular microenvironment and underlying genetic profile of the malignant cells [6,7]. For example, oxygen availability determines the rate of aerobic glycolysis utilization throughout brain cancer tumors. Matrix-assisted laser desorption/ionization (MALDI) mass spectrometry imaging (MSI) is a robust tool for studying the spatial distribution of metabolic alterations [8]. MALDI MSI rasterizes thin sections of tissue to produce mass spectra for each individual pixel, allowing for the detection of various analytes, such as small molecules, lipids, and peptides. Thus, MALDI MSI enables highly sensitive analysis with a spatial resolution ranging between 10 and 200 μm. Due to its favorable balance between the ease of sample preparation, chemical specificity/sensitivity, and spatial resolution, MALDI MSI has become the most widely used tool for spatial metabolomics in recent years.

MALDI MSI can use different analyzers, including Time-Of-Flight (TOF), Fourier transform ion cyclotron resonance, Orbitrap, and a quadrupole ion trap, among which the former is the most common. The TOF analyzer measures the time it takes for ions to travel a certain distance, which is proportional to their mass-to-charge (*m/z*) ratio, while the Fourier transform ion cyclotron resonance uses a magnetic field to separate ions based on their *m/z* ratio. While the latter provides a favorable mass resolution and mass accuracy for the unambiguous identification of molecules, MALDI-TOF MSI provides a considerably faster acquisition time (several hours for tissue sections with an area of ~1 cm^2^) and broader mass range [9]. Hence, MALDI-TOF MSI has become the prevalent technique for investigating the spatial distribution of analytes in biological samples, making it a valuable tool in the field of biomarker discovery and disease diagnosis [10].

MALDI MSI can be performed under different environmental conditions during the ionization process. Vacuum MALDI involves the ionization of the sample in a vacuum environment and is traditionally used in MALDI-TOF MSI. Recently, atmospheric-pressure MALDI has attracted much attention due to its softer ionization and more focused beams than traditional-vacuum MALDI [11,12], which help preserve the sample integrity [13]. Due to the small laser spot size, atmospheric-pressure MALDI allows us to obtain high-spatial resolution data and better reproducibility of the ions/in-source fragments across the experiment. 

The selection of a suitable matrix, which is used to assist in the desorption and ionization of the molecules from the sample surface, and deposition technique of the matrix are crucial for the success of MALDI-TOF MSI [14]. There are many matrices that are each suitable for different analytes of interest. Common considerations include (i) optical absorption [15], (ii) interference with analytes of interest, (iii) the polarity setting, (iv) stability, (v) sensitivity [16], (vi) vaporization ease [17], and (vii) the mass range of the analytes of interest [18,19,20]. Every matrix has its limitations, and knowledge regarding their characteristics is paramount in obtaining good-quality data. For example, there has been substantial evidence in the field showing the importance of crystal size and the thickness of the matrix on reproducibility and sensitivity [21,22]. Hence, currently, matrix selection still needs to be optimized for each experiment. To our best knowledge, there are no comprehensive reports comparing the numerous matrices available and their small molecules and lipid coverage on brain cancer samples in positive- and negative-ion mode by using atmospheric-pressure MALDI-TOF MSI. 

Here we compared three different matrices and two deposition techniques using both positive and negative polarities in atmospheric-pressure MALDI-TOF MSI for the detection of small molecules and lipids in murine and human brain tumors. We not only establish recommendations for matrix selection for this experimental setting but present a strategy, including robust and automated open-source computational pipelines, for the evaluation of different sample preparations applicable to any experimental setting.

## 2. Materials and Methods

### 2.1. Mouse Model

All the experiments presented in this study were conducted according to regulatory standards approved by the Walter and Eliza Hall Institute Animal Ethics Committee. The CT-2A syngeneic mouse cell line (10,000 cells) [23] was transplanted into the cortex of female 7-week-old C57Bl/6 mice according to standard procedures by using the coordinates 1 mm lateral and 1 mm anterior to the bregma to a depth of 2.5 mm [24]. The mice were monitored for neurological disturbances and collected as a cohort at an ethical endpoint. Tumors were harvested within the intact brains, transferred to the laboratory in phosphate-buffered saline (PBS), and the dried sample was immediately flash-frozen directly in isopentane in a liquid-nitrogen bath. The brains were stored at −80 °C until downstream sample preparation. Where relevant for bulk studies, tumor and normal regions were dissected directly from the frozen material on dry ice and processed independently. The tumor location was identified by a hematoxylin and eosin stain.

### 2.2. Human Samples

Patient samples were obtained directly from surgery via the Royal Melbourne Hospital Neurosurgery Brain and Spine Tumor Tissue Bank (Melbourne Health Ethics #2020.214) and were immediately flash-frozen directly in isopentane in a liquid-nitrogen bath. The patient tumors were stored at −80 °C until downstream sample preparation.

### 2.3. Sample Preparation

#### 2.3.1. MALDI-TOF-Based Mass Spectrometry Experiment

Frozen tissue was sectioned at a thickness of 10 µm directly onto Indium Tin Oxide (ITO)-coated glass slides (a surface resistance of 70–100 Ω). Optimal cutting temperature (OCT) reagent was used only to mount the tissue onto the cryotome chuck (−17 °C) for sectioning and was not exposed in the region of sectioning for metabolomics analysis due to interference. Sections were stored at −80 °C until the analysis.

Frozen sections were dried in a freeze dryer (MODULYOD, Thermo Electron Corporation, Waltham, MA, USA) for 30 min, followed by the collection of optical images by using the light microscope embedded in the MALDI-TOF MSI instrument (iMScope^TM^ QT, Shimadzu, Kyoto, Japan) prior to the matrix application. The samples that underwent spraying (2step) were dried in a vacuum desiccator for 30 min before the MSI analysis.

To compare the different matrices and application methods (Table 1, Supplementary Material Figure S1a), serial sections on separate ITO glass slides were coated with the various MALDI matrices: MALDI grade 9-Aminoacridine (9-AA, P# 92817); 2,5-Dihydroxybenzoic acid (DHB, P# 149357); and α-cyano-4-hydroxycinnamic acid (CHCA, P# C2020) purchased from Sigma-Aldrich (St Louis, MO, USA). Matrix deposition was performed either by single-step matrix vapor deposition by using iMLayer (Shimadzu, Kyoto, Japan) to sublimate and deposit an even layer of small crystals across the surface of the tissue or by a 2-step deposition method by using iMLayer for sublimation and iMLayer AERO (Shimadzu, Kyoto, Japan) for matrix spraying to recrystallize and obtain fine matrix crystals, which enable high sensitivity and a high spatial resolution (Supplementary Material Figure S1b). The thickness of the vapor-deposited matrix was 1.5, 0.9, and 0.7 µm, and the deposition temperature was 180, 220, and 250 °C for the DHB, 9-AA, and CHCA matrices, respectively. To recrystallize the matrix vapor-deposited tissue samples, 9-AA and CHCA matrix solutions were used for spraying through iMLayer AERO. Both the thickness and temperature remained the same for each of the matrices regardless of recrystallization. The 9-AA matrix concentration was kept at 10 mg/mL in a methanol/water (90:10, *v*/*v*) solution. Four layers of 9-AA were sprayed at a 90 mm/sec stage speed, with a 1 sec dry time at a 5 cm nozzle distance. The pumping pressure and the spraying pressure were kept constant at 0.05 and 0.15 MPa, respectively. For the CHCA matrix spraying, 8 layers of 10 mg/mL CHCA in acetonitrile/water (50:50, *v*/*v*) with a 0.1% trifluoroacetic acid solution were used. The stage was kept at 70 mm/sec with a 1 sec dry time at a 5 cm nozzle distance, and the pumping pressure kept was constant at 0.1 and 0.2 MPa. Recrystallized DHB was not used as it failed to form fine crystals, in line with previous reports [25].

All the MSI experiments were performed by using the iMScope^TM^ QT instrument (Shimadzu, Kyoto, Japan). The instrument is equipped with a laser-diode-excited Nd:YAG laser and an atmospheric-pressure MALDI (Shimadzu, Kyoto, Japan). It is well known for its speed, accuracy, sensitivity (ESI-positive 1 pg reserpine: S/N > 1000:1; ESI-negative 1 pg chloramphenicol: S/N > 1000:1), spatial resolution (up to 5 µm), and being user friendly [26]. To achieve better MS sensitivity, the data of the two neighboring *m/z* ranges 70–400 for polar metabolites and *m/z* 400–1000 Da for lipids with positive and negative polarity were acquired. The laser diameter (the spatial resolution) was kept at 10 µm, and the same pitch (the variable stage step down) was matched to the laser diameter. For all the samples, the data were acquired by using a laser intensity of 60 for a 10 µm spatial resolution, the detector voltage was set at 2.36 kV, the laser repetition frequency was set at 2000 Hz, the desolvation line temperature was maintained at 250 °C, and a laser irradiation count of 50 shots was accumulated per pixel.

**Table 1:** Summary of matrices, deposition methods, adduct ion, and ranges.

Matrix	Abbreviation	Deposition Method	Adduct Ion/Polarity	Ranges (*m/z*)
9-Aminoacridine	9-AA- [27]	Sublimation only	Anionized ([M^−^H] ^−^)	70–400 400–1000
9-Aminoacridine	9-AA (2step)-	Sublimation followed by recrystallization	Anionized ([M^−^H] ^−^)	70–400 400–1000
9-Aminoacridine	9-AA+	Sublimation only	Cationized ([M^+^Na]^+^; [M^+^K]^+^; [M^+^H]^+^)	70–400
9-Aminoacridine	9-AA (2step)+	Sublimation followed by recrystallization	Cationized ([M^+^Na]^+^; [M^+^K]^+^; [M^+^H]^+^)	70–400
α-cyano-4-hydroxycinnamic acid	CHCA- [28]	Sublimation only	Anionized ([M^−^H] ^−^)	70–400
α-cyano-4-hydroxycinnamic acid	CHCA (2step)-	Sublimation followed by recrystallization	Anionized ([M^−^H] ^−^)	70–400
α-cyano-4-hydroxycinnamic acid	CHCA+	Sublimation only	Cationized ([M^+^Na]^+^; [M^+^K]^+^; [M^+^H]^+^)	70–400 400–1000
α-cyano-4-hydroxycinnamic acid	CHCA (2step)+	Sublimation followed by recrystallization	Cationized ([M^+^Na]^+^; [M^+^K]^+^; [M^+^H]^+^)	70–400 400–1000
2,5- dihydroxy benzoic acid	DHB- [29]	Sublimation only	Anionized ([M^−^H] ^−^)	70–400
2,5- dihydroxy benzoic acid	DHB+	Sublimation only	Cationized ([M^+^Na]^+^; [M^+^K]^+^; [M^+^H]^+^)	70–400

#### 2.3.2. Liquid Chromatography–Mass Spectrometry

Pre-weighed tissue samples of 20 mg each were extracted for liquid chromatography–mass spectrometry (LC-MS) analysis by homogenization in a Precellys^®^ 24 Tissue homogenizer coupled to a Cryolys^®^ cooling system (Bertin Technologies) in 500 μL of 3:1 Methanol:Milli-Q water (containing internal standards). The extracts were vortexed and mixed on a thermomixer (10 min) to ensure complete metabolite extraction and then centrifuged at 4 °C for 10 min at 12,700 rpm to remove the tissue pellet.

For polar metabolite analysis: 80 μL of the methanol/water supernatant was transferred into a glass HPLC vial containing a glass insert. A further 20 μL from each sample was pooled to generate pooled biological quality control (pbQC) samples, which were run after every five biological samples. 

Analyses of the polar analytes in the samples were performed by using the Orbitrap ID-X Tribrid mass spectrometer (Thermo Scientific, Waltham, MA, USA) coupled to a Vanquish Horizon UHPLC system (Thermo Scientific, Waltham, MA, USA). The separation of polar metabolites was performed on a Merck SeQuant ZIC-HILIC column (150 mm × 4.6 mm, 5 μm particle size) maintained at 25 °C by using a binary gradient consisting of solvent A: 20 mM ammonium carbonate (pH 9.0; Sigma-Aldrich, Burlington, MA, USA) and solvent B: 100% ACN. The gradient run was as follows: time (t) = 0.0 min, 80% B; t = 0.5 min, 80% B; t = 15.5 min, 50% B; t = 17.5 min, 30% B; t = 18.5 min, 5%; t = 21.0 min, 5% B; t = 23–33 min, 80% at a solvent flow rate of 300 μL/min.

Data analysis was performed by using El Maven software (v0.12.1). Level 1 metabolite identification, according to the Metabolite Standard Initiative [30], was based on matching accurate mass and retention time to the 550 authentic standards in the Metabolomics Australia in-house library.

For the targeted lipid analysis: 600 μL of 100% chloroform (containing lipid internal standards) was added to the remaining homogenate to bring the ratio to 2:1 chloroform:methanol. The extracts were vigorously vortexed to resuspend and further extract the lipids from the tissue pellet. The samples were then mixed on a thermomixer (10 min at 10 °C) and centrifuged at 10 °C for 5 min at maximum speed to pellet the protein. The lipid extract was dried in 5 × 100 μL aliquots on a rotational vacuum concentrator (Christ RVC 2-33 CDplus) at 35 °C. The aliquots were dried and then reconstituted in 100 μL of 9:1 butanol:methanol. For a pbQC, 10 μL of each sample was pooled into a fresh vial.

Analyses of the tissue lipids were performed on the Agilent 6490 LC-QQQ mass spectrometer coupled with an Agilent 1290 series HPLC system. The separation of the lipids was conducted on a ZORBAX RRHD UHPLC C18 column (2.1 × 100 mm 1.8 mm, Agilent Technologies) maintained at 45 °C. A targeted mass spectrometry analysis was conducted in ESI-positive ion mode with dynamic scheduled multiple-reaction monitoring. The mass spectrometry settings and multiple-reaction-monitoring transitions for each lipid subclass and individual species were run as previously described [31]. Data analysis and the integration of the chromatographic peaks were performed by using MassHunter (B 10.1, Agilent Technologies, Santa Clara, CA, USA) software.

#### 2.3.3. Bulk RNA Sequencing

The extracted normal and tumor regions were ground in liquid nitrogen, and the RNA was extracted by using the RNeasy RNA extraction kit (Qiagen 74104, Qiagen, Germantown, MD, USA) and then analyzed by using a 2200 Tapestation Analyzer (Agilent, Santa Clara, CA, USA). An input of 500 ng of RNA was used to generate libraries (TruSeq RNA Library Prep v2, Illumina, San Diego, CA, USA). Sequencing was performed on the NextSeq System (Illumina) to produce 132bp single-end reads.

### 2.4. Computational Analysis

#### 2.4.1. File Conversion

MALDI-TOF MSI data were obtained in the Shimadzu IMDX format. To facilitate subsequent analytical procedures, we converted the data into ImzML format by using the “ImzML converter” (version 1.21.0 11302) functionality within the Shimadzu IMAGEREVEAL MS platform. 

#### 2.4.2. Cardinal Pipeline

**Figure 1:**
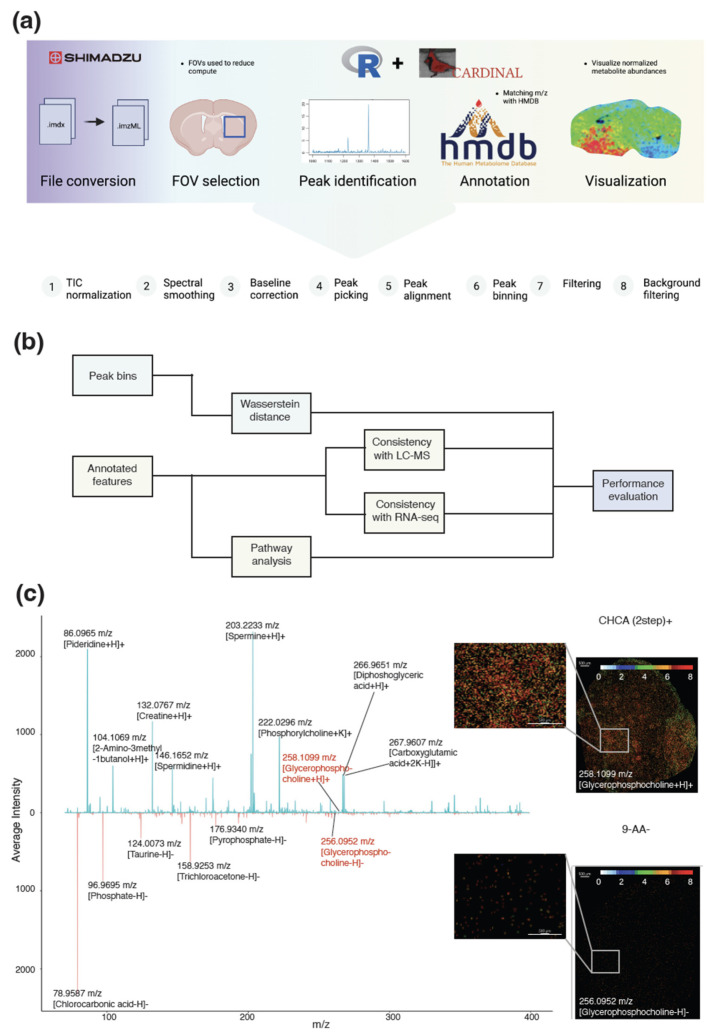
Computational pipeline. (**a**) Computational pipeline for the analysis of MALDI-TOF data, (**b**) schematic of evaluation strategy, and (**c**) comparison of spectra of small-metabolite screen by using the recrystallized CHCA matrix in positive polarity mode (**top**) and 9-AA in negative polarity mode (**bottom**) and spatial abundance of Glycerophosphocholine with enlarged areas shown in separate plots.

We used the Bioconductor Cardinal package (version 3.0.2) with R-4.3.0, a specialized tool for the manipulation of MSI data, to process all the MALDI-TOF MSI data. Our pipeline involved the following steps (Figure 1a):

- Field of View (FOV) selection;
- Spectrum acquisition and preprocessing;
- Reference peak identification and refinement;
- Spectrum binning;
- Background peak removal.

The initial spectral data were imported into the R environment as an “S4 object of class MSContinuousImagingExperiment” by using the Cardinal::readImzML function [32]. To prevent memory-related issues during processing, a FOV of size 300 pixels by 300 pixels was selected. The FOVs encompassed both the tumor region and the healthy brain region. Subsequently, normalization was performed by utilizing the total ion current (TIC) method, followed by spectrum smoothing via a Savitzky–Golay filter. Baseline distortions within the spectral data were addressed by applying median interpolation methods across 500 segments of the original spectrum.

To identify reference spectral peaks with significance, the acquired spectrum was processed by using the Cardinal::peakpick function. The alignment of the spectrum was conducted by using Cardinal::peakalign, incorporating a default estimated tolerance to yield candidate peaks. These candidate peaks were then subjected to a refinement process by using the Cardinal::peakFilter function. This involved selecting local maxima across five spectral windows while enforcing a minimum signal-to-noise ratio of 6. Additionally, a criterion was set requiring the signal to be detected in at least 1% of all pixels. The processing of the background peak bins followed the same procedures by using matrix-only sample preparations (no tissue), with the exception that peaks were selected based on a minimum detection frequency in at least 5% of the pixels. This criterion was applied because background signals tend to be less densely distributed compared to signals originating from the tissue.

Next, the processed spectrum was subjected to binning by employing the Cardinal::peakBin function on the filtered reference peaks. These peak bins encapsulated the final spectrum information. Finally, if an experimental peak is detected within 50 parts per million (ppm) representing our estimated mass resolution at its nearest background peak, then the signal peak is removed to avoid noise. These peaks were subsequently utilized for downstream analyses (Figure 1b) and annotation tasks (Figure 1c, Supplementary Material Figures S2a–c and S3a).

#### 2.4.3. Annotation of Peaks

The peaks identified by Cardinal were matched against the Human Metabolome Database (HMDB, version 5.0) [33] by using the k-nearest neighbor methodology (Figure 1c, Supplementary Material Tables S1 and S2). For each peak, a set of 30 nearest neighbors from the generated HMDB peak list is identified. To refine the selection, metabolites with distances exceeding 50 ppm from the peak bin center are filtered out. The selection of 50 ppm as the filtering threshold is underpinned by the characteristics of the Shimadzu iMScope QT mass resolution as our empirical investigation involving estimation around the peak produced by the matrix demonstrated that the average TOF resolution was approximately 50 ppm. It is important to acknowledge that a single annotation of an HMDB entry can emanate from distinct peaks, attributable to different adduct ions. 

#### 2.4.4. Pathway Analysis

We employed an overrepresentation-based pathway analysis via the stats::fisher.test (version 4.3.0) to assess the significance of the associations between the metabolite hits and reference pathways obtained from the Relational Database of Metabolomics Pathways (RaMP, database version 2.3.0) [34]. The RaMP database captures both genetic and metabolomic information linked to specific pathways. The elements within these pathways are transformed into two distinct components: a metabolomics-specific portion and a gene-specific portion. These components were then stored as individual list items within the R programming environment. The *p*-value was adjusted by the False Discovery Rate (FDR) by using Benjamini Hochberg for multiple testing.

#### 2.4.5. Kernel Density Estimation

To effectively compare the distribution of the peak bins between matrices, a comprehensive approach involving the estimation of the distribution of the peaks through the application of a Gaussian Kernel Density Estimation (KDE) was used. It is important to note that our objective here is distinct from the typical KDE approach, which aims to generate smooth curves. The computation of the KDE was performed in R by using the stats::density (version 4.3.0) function with an unbiased cross-validation bandwidth and parameter *n* = 2500 (number of returned x-y coordinates) for small molecules and *n* = 5000 for lipids. 

#### 2.4.6. Wasserstein Distance

The average intensity of a peak within a 50-pixel-by-50-pixel Region Of Interest (ROI) centered at the pathologically distinct tumor-injected region and a 50-pixel-by-50-pixel ROI within the unaffected brain region were compared by using a two-dimensional Wasserstein distance; the data were converted to an object of class “pp” with the CRAN package transport (version 0.14.6), and the Wasserstein distance was calculated by using the transport::wasserstein function.

Additionally, the Wasserstein distance was used to compare the peak bins detected in different matrices. The comparison involved assessing the KDE of the peak bin distribution across various matrices. Since the *m/z* ratio of the peaks represents diverse molecules in different ion modes, the *m/z* values of the individual coordinates used to calculate the Wasserstein distance are corrected by subtracting the adduct ion mass. Given that each matrix exhibits a distribution of densities along the *m/z* detection range, the comparison was conducted within this context. 

#### 2.4.7. Bulk RNA-Sequencing Processing

The bulk RNA-sequencing (RNA-seq) data were aligned by using STAR (version 2.7.3a) with reference to mouse mm10 genome sequences. The aligned RNA-seq data were normalized by the edgeR package (version 3.42.4) with edgeR::calcNormFactors and edgeR::cpm to obtain the normalized count [35]. 

#### 2.4.8. Comparison with LC-MS Data

The LC-MS metabolite data were preprocessed, including peak intensity normalization with the TIC method, background intensity subtraction, and annotation (Supplementary Material Figure S3b,c). A comparison of the LC-MS data with the MALDI-TOF MSI data involved the alignment of HMDB IDs, the chemical formula, and synonyms. To compute the consistency between the LC-MS and MALDI-TOF MSI data, we computed the common regulatory trend in the tumor versus the normal brain region. Specifically, in cases where a potential *m/z* value that corresponds to the HMDB ID has a regulatory trend (upregulated or downregulated in the tumor region) that is aligned with the trends observed in the LC-MS data, the metabolites associated with the HMDB ID are regarded as consistent. This strategic determination accommodates the intricate interplay of data sources and the diverse regulatory dynamics manifesting in the tumor region. Due to the absence of information about lipid backbones, we used a match of *m/z* values with a 2 and 10 ppm tolerance to align the LC-MS and MALDI-TOF MSI lipid data. 

#### 2.4.9. Comparison with RNA-Sequencing Data

To allow for the comparison between the bulk RNA-sequencing data and spatial metabolomics data while reflecting the relative regulations of specific pathways in the tumor region, rank-based pathway enrichment was utilized. The rank-based pathway enrichment was conducted by using the fgsea::fgsea (version 1.24.0) function [36]; a minimal size of sets of three was considered while the maximal size was set to 1500. The test was permuted 2500 times. Reference molecular signatures in different pathways were derived from RaMP.

## 3. Results

### 3.1. Matrix Selection for Small Molecules in Murine Glioma

**Figure 2:**
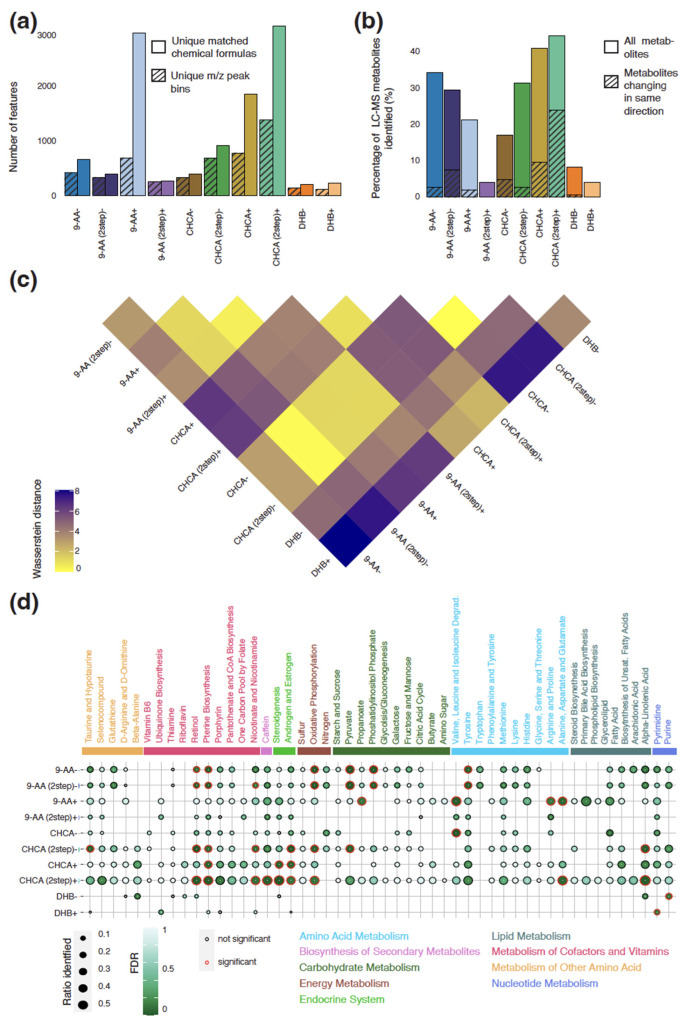
Comparison of different matrix and polarity combinations for detection of small molecules in murine glioma. (**a**) Number of unique identified annotated peaks and *m*/*z* peaks for each combination; (**b**) percentage of metabolites detected by LC-MS identified by each combination, whereby shaded area indicates the percentage of metabolites that had the same direction between the tumor and normal regions between LC-MS and MALDI-TOF; (**c**) Wasserstein distance between each combination, with higher Wasserstein distances between two combinations indicating larger differences between identified peak distribution; and (**d**) ratio of detected metabolites per KEGG pathway and associated FDR for each combination, whereby significantly enriched pathways are indicated with a red outline.

We compared the number of unique peaks detected for each matrix by using MALDI-TOF MSI in positive and negative polarity mode across both tumor and normal tissue in mice with a transplanted CT-2A glioma. We detected 1417 processed peaks by using the CHCA two-step recrystallization method in positive polarity mode followed by 801 and 709 peaks by using CHCA and 9-AA sublimation in positive polarity mode, respectively (Figure 2a). The worst performing matrix was DHB, which only detected fewer than 140 peaks in both polarities. Unsurprisingly, we also found the least number of peaks attributed to the matrix for DHB in both polarities and the most background peaks for the recrystallized CHCA matrix in positive polarity mode (Supplementary Material Figure S4a). For all matrices and in either polarity, we identified a larger number of uniquely annotated chemical structures than unique peak bins with an average of 2.16 chemical formulas (isomers share the same formula) being matched to each peak. Meanwhile, on average, 2.63 chemical formulas and 1.35 chemical formulas are matched on a single peak in positive and negative mode, respectively, owing to the relatively poor resolution of MALDI-TOF MSI.

LC-MS on both tumor and normal samples from the mice with the CT-2A glioma detected a total of 145 unique annotated small metabolites (70–400 *m/z* range). Comparing the detected peaks with MALDI-TOF MSI, we found that while the recrystallized CHCA matrix in positive polarity mode detected approximately two fold the number of raw peaks compared to CHCA sublimation only, both matrices matched approximately 40% of the LC-MS metabolites. In contrast, the 9-AA in positive polarity mode, which had the second-highest number of raw peaks detected, only identified 34% of the LC-MS metabolites. 9-AA using both deposition methods in positive polarity mode, along with DHB in both polarities, also identified a few metabolites that had been present in previous studies of brain cancer (Supplementary Material Figure S4b). Recrystallized CHCA, in both polarities, as well as 9-AA using both deposition methods in negative polarity mode detected 13 of 15 metabolites of interest that were detected in previous studies of brain cancer [37,38].

The 9-AA matrices using both polarities identified similar spectra (Figure 2c). These spectra were similar to those found by the CHCA matrices (using both deposition techniques) in negative polarity mode. CHCA matrices in positive polarity mode and DHB matrices in both polarities identified spectra that were more dissimilar to the spectra identified by other matrices and polarity combinations and each other. Consistent with the dissimilarity between the spectra produced by DHB compared to other sample preparations and polarities, peaks with annotatable entries in the HMDB identified with the DHB matrix in negative polarity mode were largely not detected by other matrix and polarity combinations (Supplementary Material Figure S4c). In contrast, 71% of the matched chemical formulas identified by the recrystallized CHCA matrix in positive polarity mode were also found by other matrix and polarity combinations, possibly owing to the large total number of identified peaks. Interestingly, for 9-AA in positive polarity mode, 18% of all the matched chemical formulas were exclusively detected despite the low overall number of peaks detected.

To assess whether the different matrix and polarity combinations detected similar biological programs, we conducted an overrepresentation-based pathway analysis. In general, most matrix and polarity combinations, apart from those involving DHB, detected metabolites in the same KEGG pathways (Figure 2d). Purine metabolites, indicative of dividing cells and therefore crucial to examine cancer growth, were completely undetected in several matrix and polarity combinations, including 9-AA in positive polarity mode. Furthermore, only CHCA, using both deposition techniques, in positive polarity mode showed good coverage of the metabolic pathways related to cofactors and vitamins. Pathway enrichment across multiple pathway databases indicated that the recrystallized CHCA matrix in positive polarity mode was biased towards detecting metabolic pathways related to hormone- and fatty-acid-mediated insulin secretion, amino acid metabolism and catabolism, nucleotide biosynthesis and metabolism, and sulfur amino acid metabolism (Supplementary Material Figure S4d). Interestingly, given the sample origin, there was no bias for detecting metabolic pathways related to synaptic neurotransmission for this matrix and polarity combination.

### 3.2. Matrix Selection for Lipid Detection in Murine CT-2A Glioma

**Figure 3:**
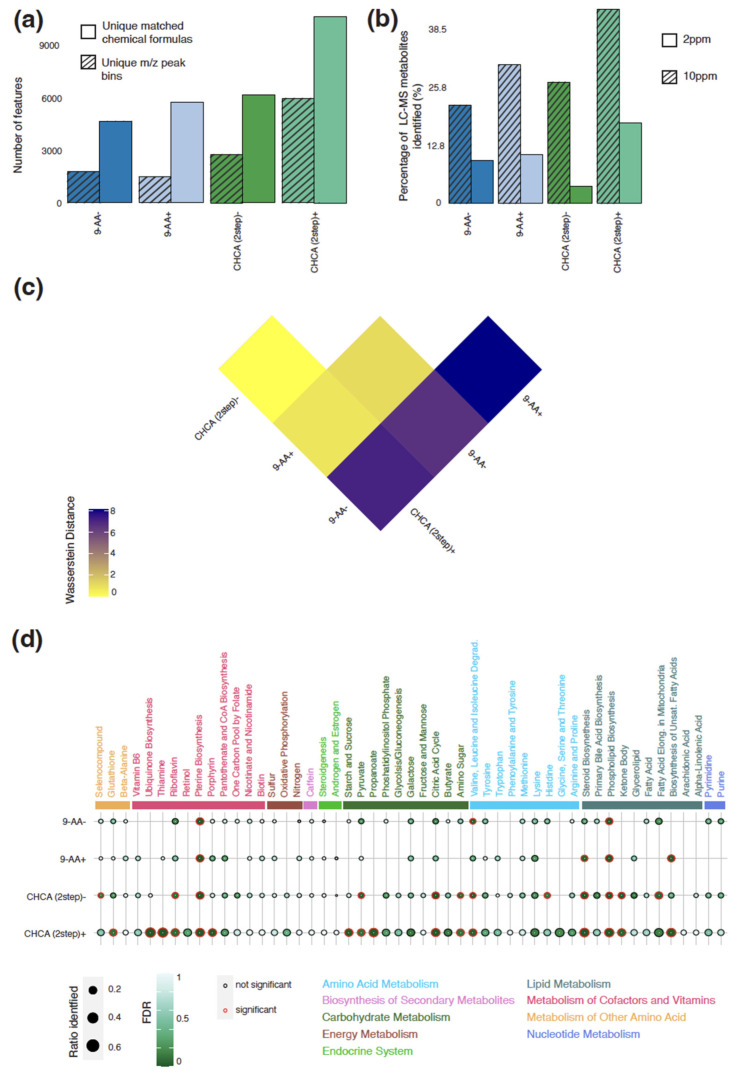
Comparison of different matrix and polarity combinations for detection of lipids in murine glioma. (**a**) Number of unique identified annotated peaks and *m/z* peaks for each combination. (**b**) Percentage of metabolites detected by LC-MS identified by each combination at 2 and 10 ppm thresholds. (**c**) Wasserstein distance between each combination with higher Wasserstein distances between two combinations indicating larger differences between identified peak distribution. (**d**) Ratio of detected metabolites per KEGG pathway and associated FDR for each combination; significantly enriched pathways are indicated with a red outline.

To assess the utility of different matrices for lipid analysis, using only the 9-AA and CHCA matrices, we first compared the total number of unique peaks detected for each tested matrix and polarity combination (an *m/z* screening range between 400 and 1000). We again detected the largest number of peaks (5959) with the recrystallized CHCA matrix in positive polarity mode (Figure 3a). We also found the largest number of peaks attributed to this matrix and polarity combination (Supplementary Material Figure S5a). Out of all the tested matrix and polarity combinations, 9-AA in negative polarity mode detected the least number of total and background peaks. For all the matrices and in either polarity, we identified a much larger number of unique annotated metabolites than unique peaks, whereby on average 2.26 chemical formulas were matched to a peak.

LC-MS on both tumor and normal samples from the mice with a CT-2A glioma detected a total of 156 unique annotated lipids. Due to the presence of structural isomers in the lipids, the annotation of an *m/z* value to a specific structure is difficult without fragmentation patterns. To align the LC-MS and MALDI-TOF MSI data, we matched peaks detected spatially with the candidate metabolites detected by LC-MS by using two different tolerance ranges at 2 and 10 ppm. At 2 ppm, the recrystallized CHCA in negative polarity mode had the lowest overlap with less than 10% (Figure 3b). Maintaining a consistent matching ratio hierarchy, increasing the mass tolerance to 10 ppm significantly increased the matching ratio of the recrystallized CHCA matrix in negative polarity mode and recrystallized 9-AA in positive polarity mode, possibly due to the proton donor and receiver properties of CHCA and 9-AA, respectively. Hence, using CHCA in negative polarity mode and 9-AA in positive polarity mode might introduce extra noise. Moreover, a recrystallized CHCA matrix in positive polarity mode had the highest number of overlapping peaks at both 2 and 10 ppm owing to the total number of detected peaks. 

All matrix and polarity combinations, apart from 9-AA in negative polarity mode, identified similar spectra (Figure 3c). In line with this, 75% of the total number of matched chemical formulas across all matrix and polarity combinations were detected multiple times (Supplementary Material Figure S5b). Owing to the large number of total peaks detected, only the recrystallized CHCA matrix in positive polarity mode found more than 23% of peaks exclusively.

A pathway analysis was conducted to assess whether the different matrix and polarity combinations converged on the detection of similar biological programs. Very similar KEGG pathways were detected across all the tested matrix and polarity combinations (Figure 3d). These pathways were largely the same as those detected by the small-metabolite screen owing to the involvement of both lipids and small molecules in most pathways. As expected, the lipid screen did detect more pathways classified as lipid metabolism and was biased towards the detection of such pathways. Pathway enrichment across multiple pathway databases indicated that only the recrystallized CHCA matrix in positive polarity mode was biased towards detecting metabolic pathways related to ERBB Family Signaling, which is a key driver pathway in cancer [39] (Supplementary Material Figure S5c).

### 3.3. Differential Abundance between Tumor and Normal Regions in Murine CT-2A Glioma

**Figure 4:**
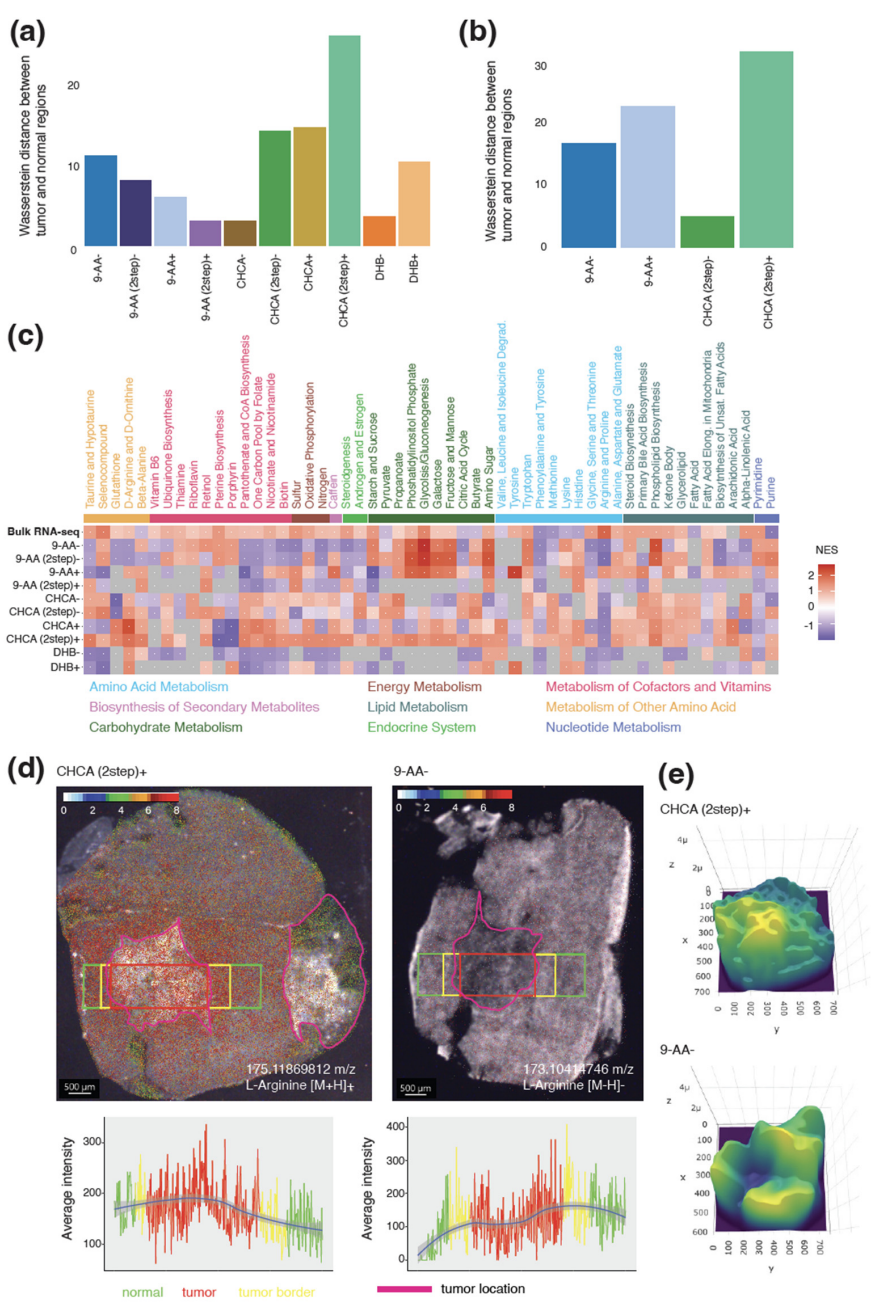
Altered metabolism between tumor and normal regions in murine glioma. (**a**) Wasserstein distance between *m/z* peak distributions of tumor and normal regions for each combination in small-molecule screen. (**b**) Wasserstein distance between *m/z* peak distributions of tumor and normal regions for each combination in lipid screen. (**c**) Normalized enrichment score for KEGG pathways for each combination and bulk RNA-seq data of tumor and normal regions for small-molecule screen. (**d**) Abundance of L-Arginine across tumor (red rectangle), border (yellow rectangle), and adjacent normal (green rectangle) as detected by recrystallized CHCA in positive polarity mode on the left and 9-AA in negative polarity mode on the right; the line plot underneath shows the average intensity across the different areas. (**e**) Three-dimensional plot of the abundance of L-Arginine for recrystallized CHCA matrix in positive polarity mode (**top**) and 9-AA matrix in negative polarity mode (**bottom**).

We next investigated the metabolic differences between healthy and tumor regions in the brain of mice with a transplanted CT-2A glioma. Overall, we identified the largest differences between the regions by using the recrystallized CHCA matrix in positive polarity mode for both small molecules and lipids (Figure 4a,b). Despite detecting many peaks across both ranges, the 9-AA matrix in negative polarity mode identified considerably fewer differences between the regions than the recrystallized CHCA matrix in positive polarity mode. Interestingly, the largest contribution to the differences detected in the recrystallized CHCA matrix in positive polarity mode for small molecules was spermidine, contributing more than 4% to the overall Wasserstein distance (Supplementary Material Figure S6a). Spermidine has the capacity to improve glucose and lipid metabolism [40], contributing to the promotion of tumor growth. For lipids, no single peak contributed more than 1% to the overall Wasserstein distance (Supplementary Material Figure S6b).

For the small molecules, we compared the direction of the differential abundance for each matrix and polarity combination (Figure 2b). Of the metabolites identified, 54% shared the same direction for the differential abundance between the tumor and normal regions in both the recrystallized CHCA matrix in positive polarity mode and LC-MS. For all other matrix and polarity combinations, this was below 25%. At closer inspection, the recrystallized CHCA matrix in positive polarity mode agreed with LC-MS for both reduced and increased metabolites (Supplementary Material Figure S6c). In contrast, the 9-AA matrix in negative polarity mode barely identified any of the same downregulated metabolites as LC-MS (Supplementary Material Figure S6d).

To further validate our findings, we also conducted RNA-seq on normal and tumor regions from a brain with a transplanted CT-2A glioma. To compare the results from RNA-seq to those of MSI, we calculated the normalized enrichment score for the KEGG pathways (Figure 4c and Supplementary Material Figure S6e). As for LC-MS, the recrystallized CHCA matrix in positive polarity mode was the most concordant with the RNA-seq results, with 66% of the pathways sharing the direction of the overall activity change in the pathway for small molecules and 73% for lipids. While the 9-AA matrix in negative polarity mode displayed agreement with the LC-MS results, there was less stringency with the RNA-seq results, with 26% of the pathways displaying the same overall direction in small metabolites and 30% of the pathways displaying the same overall direction in lipids. Particularly, all the KEGG pathways classified as energy metabolism and endocrine systems showed opposite direction changes between tumor and normal regions.

Examining the distribution of single metabolites across the tissue revealed stark contrasts between the 9-AA in negative polarity mode and the recrystallized CHCA matrix in positive polarity mode. L-Arginine, a conditionally essential amino acid required by cancer cells for rapid growth, would be expected to be increased in abundance in the tumor regions. Strikingly, L-Arginine was only detected at high levels by using a recrystallized CHCA matrix in positive polarity mode and was infrequently detected with the 9-AA matrix in negative polarity mode (Figure 4d). Measuring the abundance of L-Arginine spatially across the brain revealed the expected increase in the abundance of the amino acid specifically in the tumor region (Figure 4e), while 9-AA in negative polarity mode was significantly diminished across the tumor region. Interestingly, the LC-MS data only showed a negligible difference in the abundance of this metabolite between the tumor and normal regions (Supplementary Material Figure S3c). Thus, the recrystallized CHCA matrix in positive polarity mode appears to be most suited to the normal and tumor brain microenvironment. 

### 3.4. Matrix Selection for Metabolite Detection in Human IDH-Mutant Glioma

**Figure 5:**
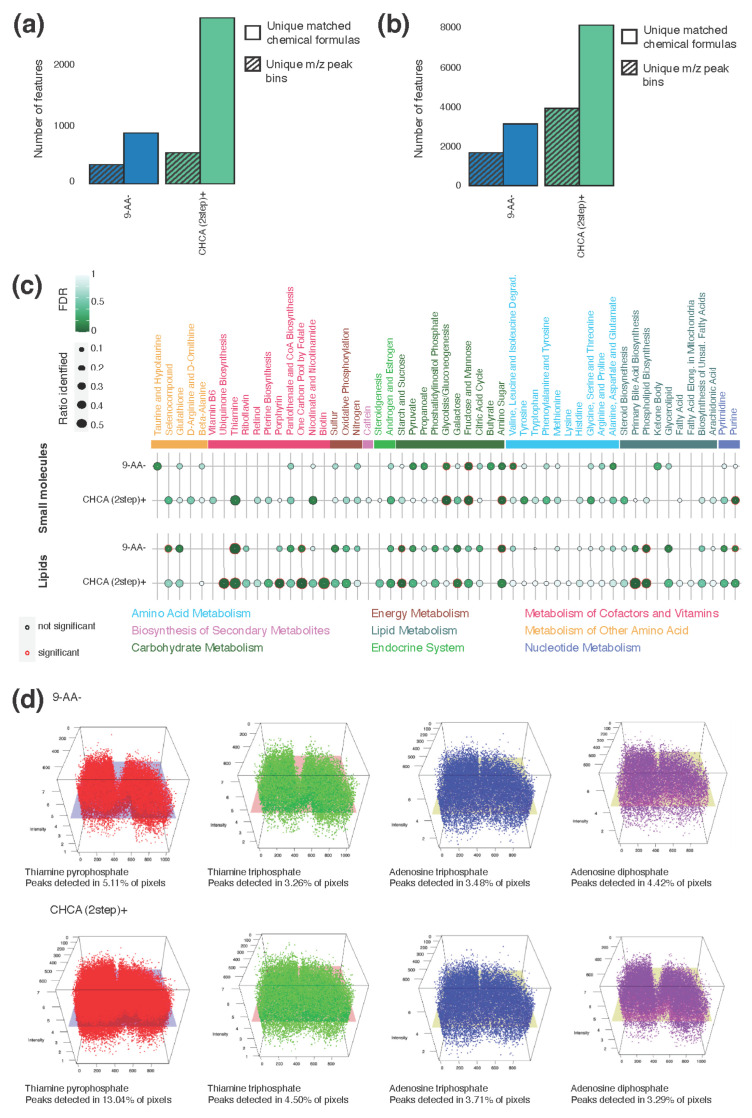
Lipid and small molecules in a tumor from a patient with *IDH1*-mutated glioma. (**a**) Number of unique identified annotated peaks and *m/z* peak bins for each combination in small-molecule screen. (**b**) Number of unique identified annotated peaks and *m/z* peak bins for each combination in lipid screen. (**c**) Ratio of detected metabolites per KEGG pathway and associated FDR for each combination separated by screen; significantly enriched pathways are indicated with a red outline. (**d**) Three-dimensional visualization of selected metabolites in the Thiamine pathway across the tumor section for 9-AA matrix in negative polarity mode (**top**) and recrystallized CHCA matrix in positive polarity mode (**bottom**) in the lipid screen.

To test whether our findings were transferable across species and brain tumor subtype, we applied the two best performing matrix and polarity combinations on a resected tumor sample from a patient with the *IDH*-mutant glioma. We found similar trends with regard to the number of detected peak bins and annotated chemical formulas, as was observed for the murine CT-2A glioma for both small metabolites and lipids (Figure 5a,b). The recrystallized CHCA matrix in positive polarity mode detected 1.64 times more peak bins for the small molecules and 2.34 times more for the lipids than the 9-AA matrix in negative polarity mode. This resulted in 227% more chemical formulas for the small molecules and 160% more for the lipids detected in the recrystallized CHCA matrix in positive polarity mode compared to the 9-AA matrix in negative polarity mode.

As previously performed, we conducted a pathway analysis for both lipids and small molecules with very similar results in terms of detection (Figure 5c). For both, most pathways represented in the 9-AA matrix in negative polarity mode were represented in the recrystallized CHCA matrix in positive polarity mode except for two lipid pathways and Butyrate in the small-molecule screen. However, the 9-AA matrix in negative polarity mode missed several pathways belonging to the group of the metabolism of cofactors and vitamins, which is required for chemical reactions in the cell. In contrast to the results observed for the murine CT-2A glioma, the recrystallized CHCA matrix in positive polarity mode was enriched for the metabolites in pathways grouped as carbohydrate metabolism rather than energy metabolism. This is likely a reflection of the biology of decreased glucose uptake in *IDH*-mutant tumors compared to *IDH*-wildtype tumors, such as the CT-2A glioma [41].

Finally, we visualized the metabolites of the Thiamine pathway, which was statistically significantly enriched in both matrices with lipids (Figure 5d), and we observed that for most metabolites enriched in the pathway, the recrystallized CHCA matrix in positive polarity mode was detectable across many more pixels than the 9-AA matrix in negative polarity mode. Furthermore, the average intensity of the metabolites was higher. 

## 4. Discussion

The selection of the matrix mixture, deposition technique, and polarity setting has a crucial impact on metabolite detection in atmospheric-pressure MALDI-TOF MSI. To guide matrix selection for the study of brain cancers, we tested several combinations of matrices, deposition techniques, and polarity settings across brain cancer types and species for both small molecules and lipids. We found that the use of the recrystallized CHCA matrix in positive polarity mode for the atmospheric-pressure MALDI-TOF MSI in the glioma allowed us to detect the greatest number of metabolites across species and brain cancer types. A comparison with the LC-MS data generated from the same samples also showed the greatest overlap for this matrix and polarity combination. Interestingly, we found that the commonly applied 9-AA matrix in negative polarity mode in the glioma setting had an inferior performance in terms of the number of metabolites detected and matching with the LC-MS data. The DHB matrix in both polarity modes performed poorly. This confirms earlier comparisons of matrices for the MALDI MSI of normal brain tissue [13] and points to experimental optimization for brain tissue being applicable to glioma tissue. This poor performance is possibly due to DHB producing large spot sizes [42].

The superiority of the recrystallized CHCA matrix in positive polarity mode among the tested combinations was also confirmed when comparing the differential abundance between tumor and normal regions across different technologies. In particular, a high degree of concordance was observed between the metabolite peaks detected by using the recrystallized CHCA matrix in positive polarity mode and RNA expression derived from RNA-seq data, increasing confidence in the results. Additionally, an example of the spatial abundance of L-Arginine, an important metabolite involved in cancer growth, revealed the sparsity of detection and reduced signal intensity in the commonly applied 9-AA matrix in negative polarity mode compared to the recrystallized CHCA matrix in positive polarity mode. A reduction in the signal by using the 9-AA matrix had been previously observed when studying gangliosides in normal brain tissue when compared to other matrices [43]. 

Our work is limited by the small number of matrices investigated. Future work should investigate other commonly applied matrices used to generate spatial metabolomics data from normal brain tissue and/or brain tumor tissue. This should include the 4-maleicanhydridoproton sponge (MAPS) matrix, which has been successfully employed to obtain ionic maps of lactate, 2-hydroxyglutarate, and chloride anions, important metabolites of interest [44]. Matrices, such as 1,5-diaminonapthalene (DAN), which was used to obtain spatial metabolomics followed by immunohistochemistry on rat brain sections [45], and 2,4,6-trihydroxyacetophenone (THAP) [13], which performed well in a recent evaluation on normal brain tissue of six different matrices, should also be evaluated in future comparative studies for their suitability for the analysis of metabolic activity in brain cancer. Additionally, future studies should test matrices with additives, such as ionic liquid matrices, that are known to improve MALDI analysis [46,47]. We recommend that future comparative studies follow our experimental and computational workflow to evaluate the performance of different matrices and deposition techniques. This workflow includes ascertaining gross differences between matrices, the biological interpretation of differences, and comparison with other techniques such as LC-MS and RNA-seq. In particular, the comparison with other techniques allows for the assessment of the overlap between results, with a greater overlap likely representing a greater number of real observed differences, thus providing an objective quality measure. Applications of our workflow will enable objective matrix selection and improve MALDI MSI for any spatial metabolomics studies in the future.

## 5. Conclusions

Methods such as atmospheric-pressure MALDI-TOF MSI have the capability to detect alterations in metabolic activity attributed to cancer. However, the quality of the data obtained can be heavily influenced by the preparation of the samples. In our study, we investigated five distinct sample-preparation protocols by using three different matrices, with a specific focus on examining brain tumor tissue for small-molecule and lipid analysis. We evaluated these protocols based on their ability to detect a broad range of metabolites, represent pathways, and their consistency when compared to other analytical techniques.

Each matrix exhibited unique strengths and weaknesses. Understanding the characteristics of these matrices is pivotal when choosing the most suitable one for the specific scientific requirements of a given study. We recommend the utilization of recrystallized CHCA to achieve high-resolution spatial imaging in positive-ion mode when analyzing brain tumor tissue through atmospheric-pressure MALDI-TOF MSI. This recommendation aligns with prior research that has also advocated for the use of this matrix when studying normal brain tissue [13].

## Data Availability

Raw data are available at www.ebi.ac.uk/metabolights/MTBLS8809 or upon request from authors. All the code is available via https://github.com/BCRL-tylu/Matrix-optimisation.

## Supplementary Materials

The following supporting information can be downloaded at https://www.mdpi.com/article/10.3390/metabo13111139/s1: Figure S1: Experimental designs; Figure S2: Spectra for atmospheric-pressure MALDI-TOF MSI; Figure S3: Spectra for human samples MS and chromatograms for LC-MS; Figure S4: Further comparisons between matrix and polarity combinations for MALDI-TOF for small molecules; Figure S5: Further comparisons between matrix and polarity combinations for MALDI-TOF for lipids; Figure S6: Extended differential metabolite abundance between tumor and normal regions; Table S1: Annotated features for small molecules detected by each sample preparation; Table S2: Annotated features for lipids detected by each sample preparation.
