# Supplementary material for: Matrix Selection for the Visualization of Small Molecules and Lipids in Brain Tumors Using Untargeted MALDI-TOF Mass Spectrometry Imaging
Source: Metabolites. 2023 Nov 9;13(11):1139. doi: 10.3390/metabo13111139 (PMC10673325; doi:10.3390/metabo13111139)
Supplement: Supplementary file 1 [file metabolites-13-01139-s001.zip › Supps/SuppFigures_comb.pdf]

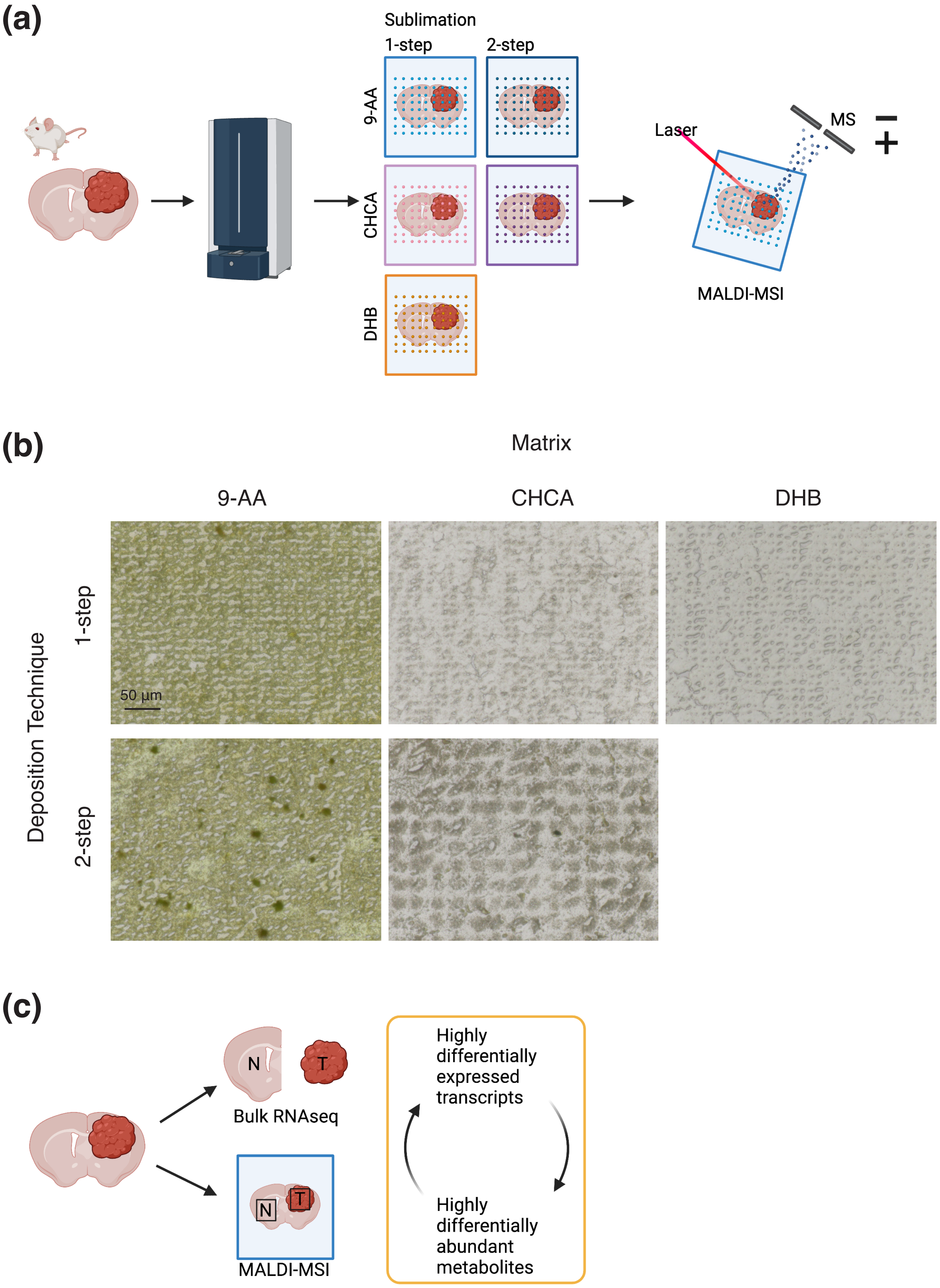
(a)
Sublimation
1-step
2-step
9-AA
CHCA
DHB
Laser
MS
MALDI-MSI
(b)
Matrix
9-AA
CHCA
DHB
Deposition Technique
1-step
2-step
50 µm
(c)
N
T
Bulk RNAseq
MALDI-MSI
Highly differentially expressed transcripts
Highly differentially abundant metabolites

Supplementary Material Figure S1. Experimental designs. (**a**) Experimental design for matrix comparison with CT-2A tumors; (b) Microscopy of MALDI-analyzed CT-2A tumors highlighting laser destruction of tissue; scale, 50 μm; (**c**) Experimental design for validation of MALDI-TOF using bulk RNA-seq.

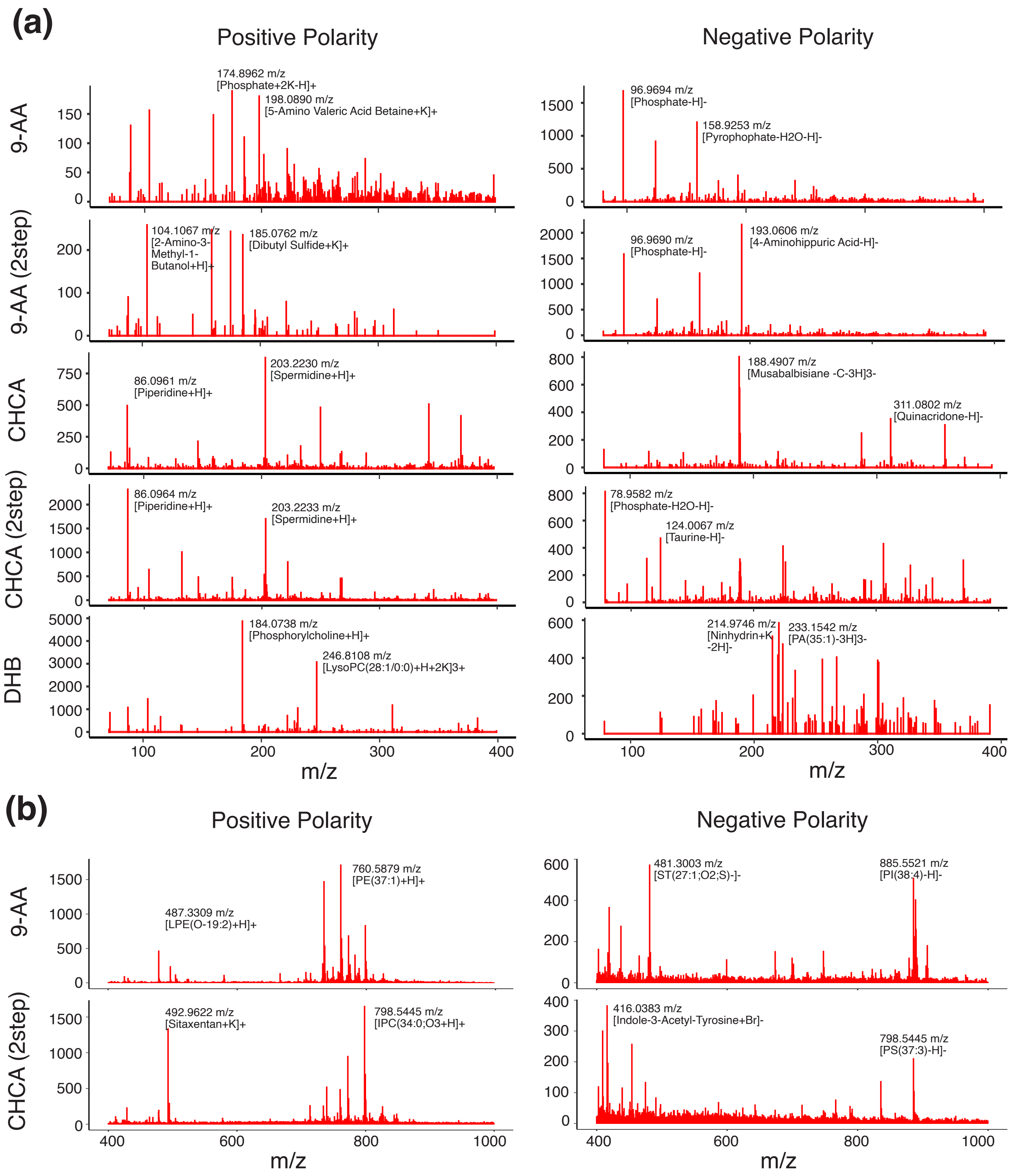

(a)
Positive Polarity
Negative Polarity
9-AA
9-AA (2step)
CHCA
CHCA (2step)
DHB
174.8962 m/z [Phosphate+2K-H]+
198.0890 m/z [5-Amino Valeric Acid Betaine+K]+
104.1067 m/z [2-Amino-3-Methyl-1-Butanol+H]+
185.0762 m/z [Dibutyl Sulfide+K]+
86.0961 m/z [Piperidine+H]+
203.2230 m/z [Spermidine+H]+
86.0964 m/z [Piperidine+H]+
203.2233 m/z [Spermidine+H]+
184.0738 m/z [Phosphorylcholine+H]+
246.8108 m/z [LysoPC(28:1/0:0)+H+2K]3+
96.9694 m/z [Phosphate-H]-
158.9253 m/z [Pyrophophate-H2O-H]-
96.9690 m/z [Phosphate-H]-
193.0606 m/z [4-Aminohippuric Acid-H]-
188.4907 m/z [Musabalbisiane -C-3H]3-
311.0802 m/z [Quinacridone-H]-
78.9582 m/z [Phosphate-H2O-H]-
124.0067 m/z [Taurine-H]-
214.9746 m/z [Ninhydrin+K -2H]-
233.1542 m/z [PA(35:1)-3H]3-
m/z
(b)
Positive Polarity
Negative Polarity
9-AA
CHCA (2step)
487.3309 m/z [LPE(O-19:2)+H]+
760.5879 m/z [PE(37:1)+H]+
492.9622 m/z [Sitaxentan+K]+
798.5445 m/z [IPC(34:0;O3+H]+
481.3003 m/z [ST(27:1;O2;S)-]-
885.5521 m/z [PI(38:4)-H]-
416.0383 m/z [Indole-3-Acetyl-Tyrosine+Br]-
798.5445 m/z [PS(37:3)-H]-
m/z

Supplementary Material Figure S2. Spectra for atmospheric-pressure MALDI-TOF MSI. (**a**) Spectra obtained for each sample preparation in small molecule screen with CT-2A glioma using atmospheric-pressure MALDI-TOF MSI; (**b**) Spectra obtained for each sample preparation in lipid screen with CT-2A glioma using atmospheric-pressure MALDI-TOF MSI.

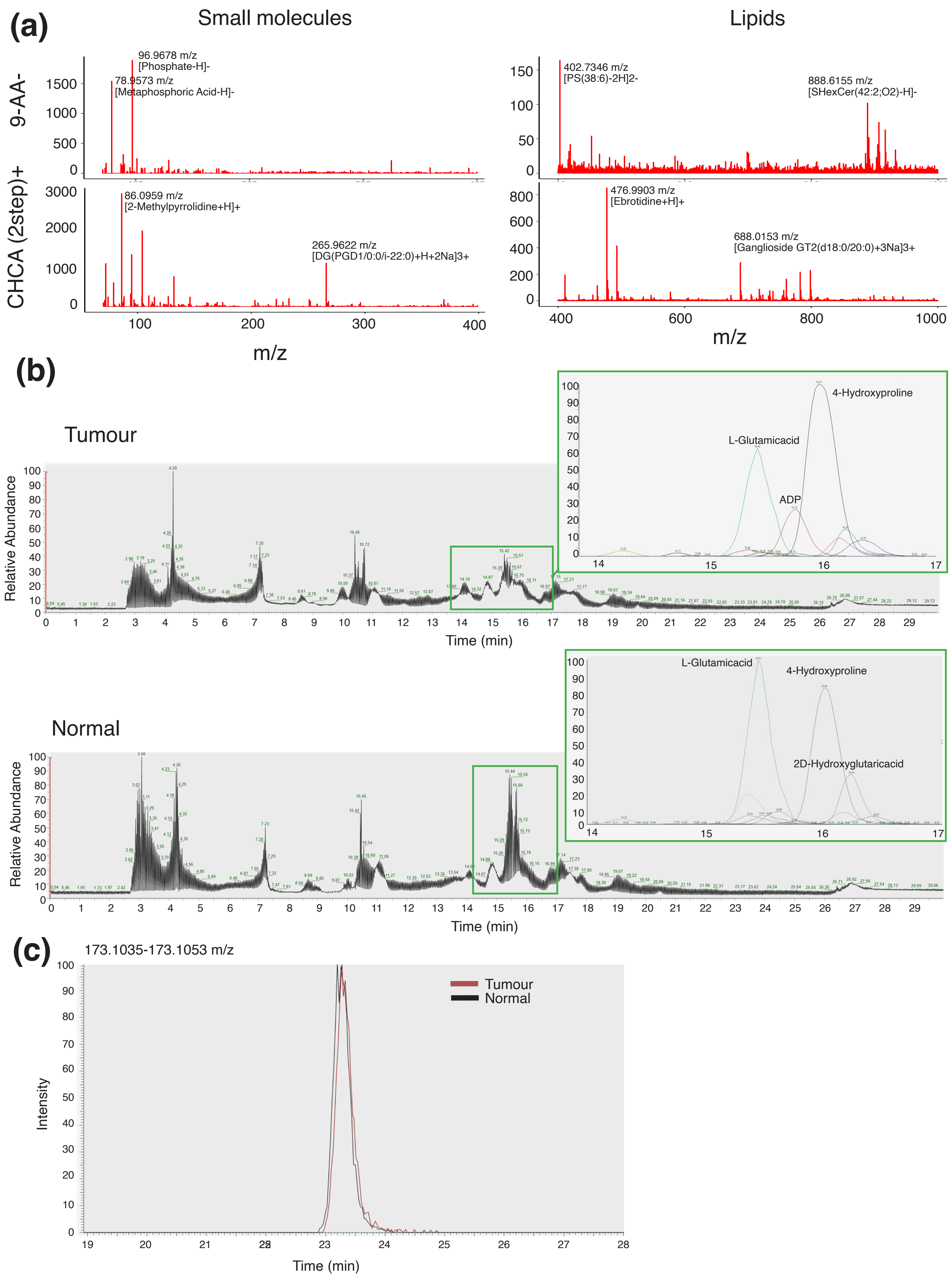

(a)
Small molecules
Lipids
9-AA-
CHCA (2step)+
96.9678 m/z
[Phosphate-H]-
78.9573 m/z
[Metaphosphoric Acid-H]-
402.7346 m/z
[PS(38:6)-2H]2-
888.6155 m/z
[SHexCer(42:2;O2)-H]-
86.0959 m/z
[2-Methylpyrrolidine+H]+
265.9622 m/z
[DG(PGD1/0:0/i-22:0)+H+2Na]3+
476.9903 m/z
[Ebrotidine+H]+
688.0153 m/z
[Ganglioside GT2(d18:0/20:0)+3Na]3+
m/z
(b)
Tumour
Normal
Relative Abundance
Time (min)
L-Glutamicacid
4-Hydroxyproline
ADP
2D-Hydroxyglutaricacid
(c)
173.1035-173.1053 m/z
Tumour
Normal
Intensity
Time (min)

Supplementary Material Figure S3. Spectra for human samples and chromatograms for LC-MS. (**a**) Spectra for human samples obtained for each sample preparation in small molecules screen (left) and lipid screen (right); (**b**) Top shows chromatogram for tumor sample bottom shows chromatogram for normal sample as obtained with LC-MS; (**c**) Extracted ion chromatogram for Arginine.

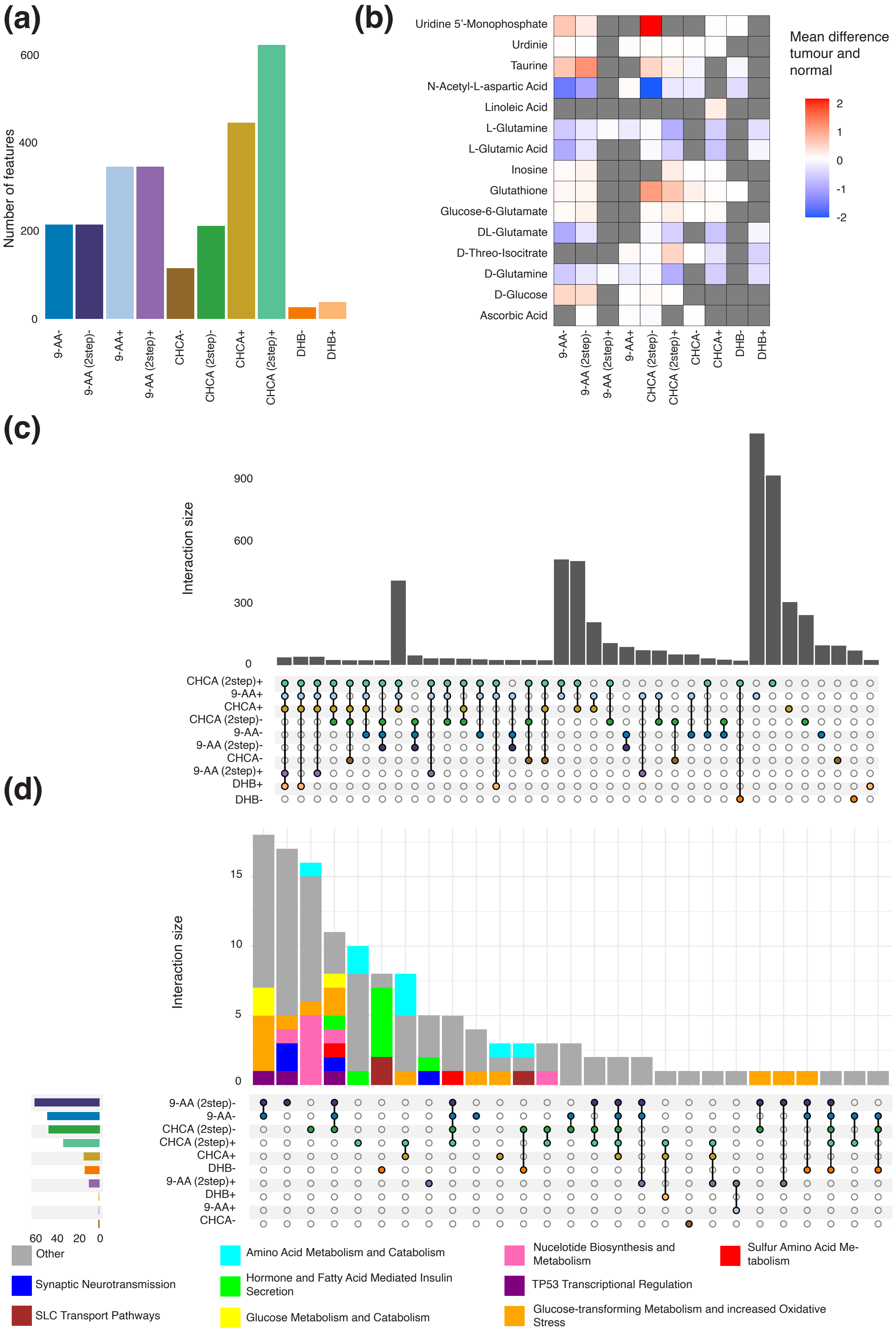
(a)
Number of features
0
200
400
600
9-AA-
9-AA (2step)-
9-AA+
9-AA (2step)+
CHCA-
CHCA (2step)-
CHCA+
CHCA (2step)+
DHB-
DHB+
(b)
Uridine 5'-Monophosphate
Urdinie
Taurine
N-Acetyl-L-aspartic Acid
Linoleic Acid
L-Glutamine
L-Glutamic Acid
Inosine
Glutathione
Glucose-6-Glutamate
DL-Glutamate
D-Threo-Isocitrate
D-Glutamine
D-Glucose
Ascorbic Acid
9-AA-
9-AA (2step)-
9-AA (2step)+
9-AA+
CHCA (2step)-
CHCA (2step)+
CHCA-
CHCA+
DHB-
DHB+
Mean difference tumour and normal
2
1
0
-1
-2
(c)
Interaction size
0
300
600
900
CHCA (2step)+
9-AA+
CHCA+
CHCA (2step)-
9-AA-
9-AA (2step)-
CHCA-
9-AA (2step)+
DHB+
DHB-
(d)
Interaction size
0
5
10
15
9-AA (2step)-
9-AA-
CHCA (2step)-
CHCA (2step)+
CHCA+
DHB-
9-AA (2step)+
DHB+
9-AA+
CHCA-
60
40
20
0
Other
Amino Acid Metabolism and Catabolism
Nucelotide Biosynthesis and Metabolism
Sulfur Amino Acid Metabolism
Synaptic Neurotransmission
Hormone and Fatty Acid Mediated Insulin Secretion
TP53 Transcriptional Regulation
SLC Transport Pathways
Glucose Metabolism and Catabolism
Glucose-transforming Metabolism and increased Oxidative Stress

Supplementary Material Figure S4. Further comparisons between matrix and polarity combinations for MALDI-TOF for small molecules. (**a**) Number of unique background peaks for each combination in small molecule screen; (**b**) Mean difference in abundance of metabolites of interest between tumor and normal regions with grey color representing metabolites not detectable; (**c**) Upset plot representing a Venn diagram of sizes of overlaps between detectable chemical formulas for different sets of matrix and polarity combinations; (**d**) Upset plot representing a Venn diagram of sizes of overlaps between enriched pathways for different sets of matrix and polarity combinations colored by semantic groups of pathways.

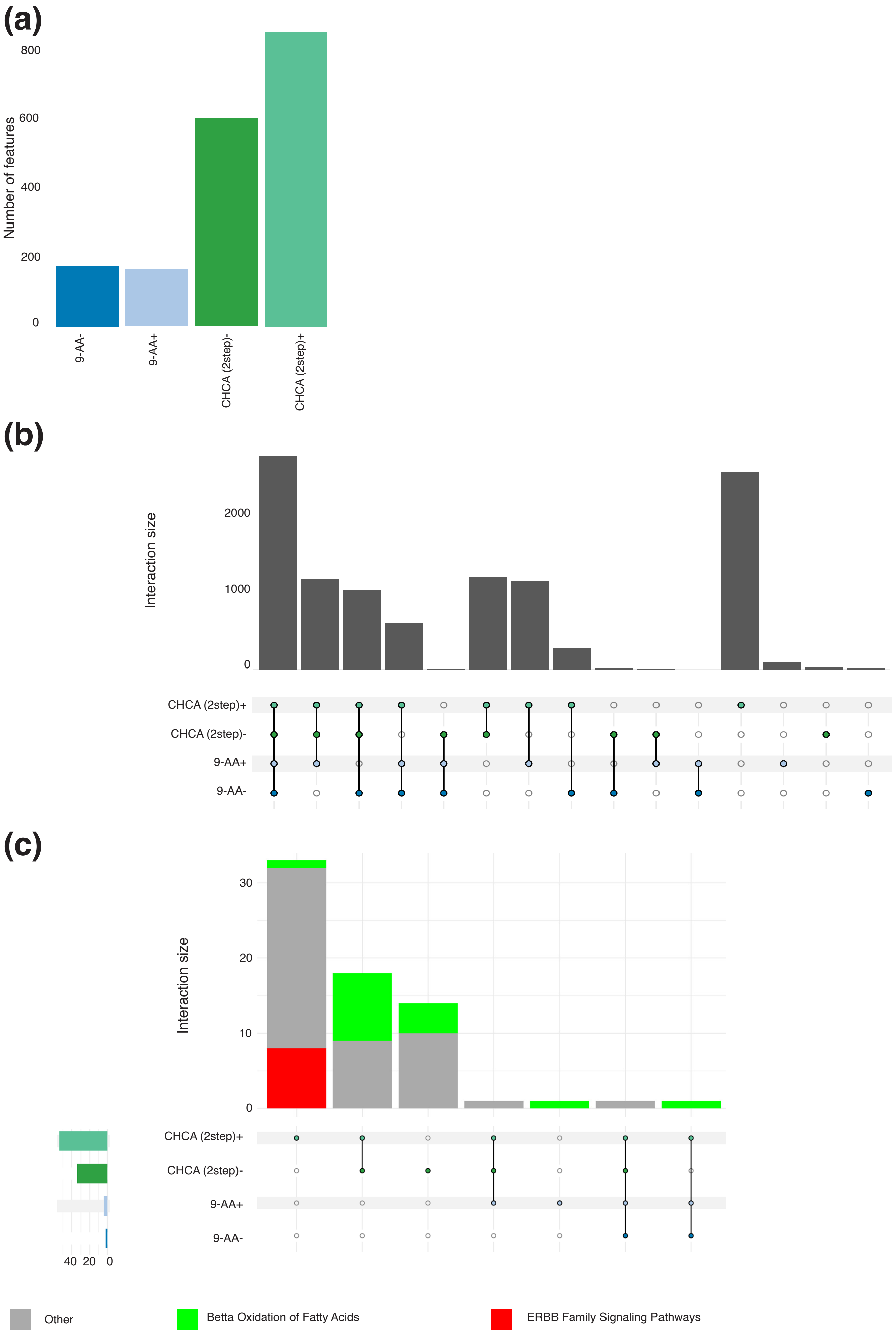
(a)
Number of features
0
200
400
600
800
9-AA-
9-AA+
CHCA (2step)-
CHCA (2step)+
(b)
Interaction size
0
1000
2000
CHCA (2step)+
CHCA (2step)-
9-AA+
9-AA-
(c)
Interaction size
0
10
20
30
CHCA (2step)+
CHCA (2step)-
9-AA+
9-AA-
40 20 0
Other
Betta Oxidation of Fatty Acids
ERBB Family Signaling Pathways

Supplementary Material Figure S5. Further comparisons between matrix and polarity combinations for MALDI-TOF for lipids. (**a**) Number of unique background peaks for each combination in small molecule screen; (**b**) Upset plot representing a Venn diagram of sizes of overlaps between detectable chemical formulas for different sets of matrix and polarity combinations; (**c**) Upset plot representing a Venn diagram of sizes of overlaps between enriched pathways for different sets of matrix and polarity combinations colored by semantic groups of pathways.

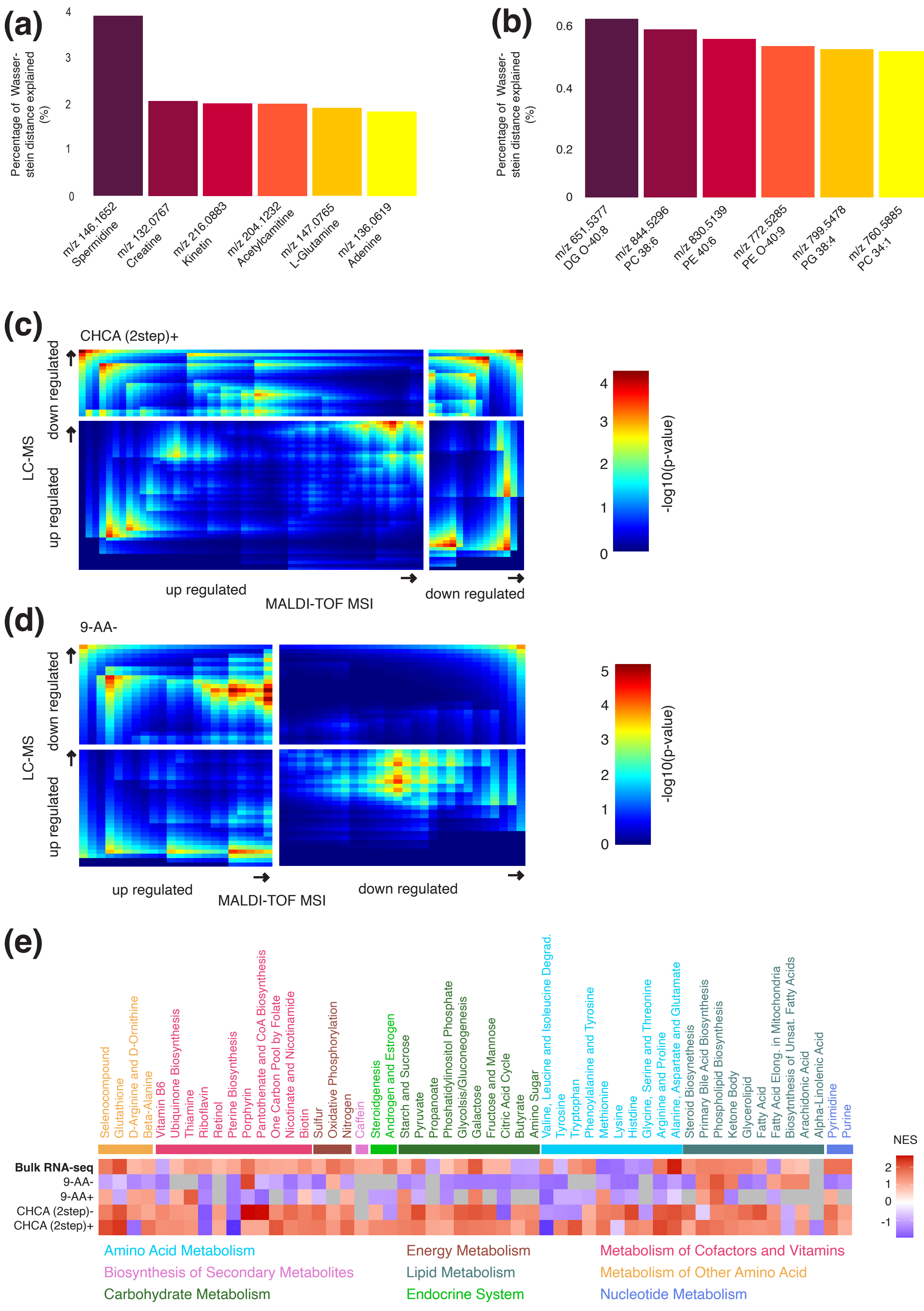
(a)
Percentage of Wasser-stein distance explained (%)
m/z 146.1652 Spermidine
m/z 132.0767 Creatine
m/z 216.0883 Kinetin
m/z 204.1232 Acetylcarnitine
m/z 147.0765 L-Glutamine
m/z 136.0619 Adenine
(b)
m/z 651.5377 DG O-40:8
m/z 844.5296 PC 38:6
m/z 830.5139 PE 40:6
m/z 772.5285 PE O-40:9
m/z 799.5478 PG 38:4
m/z 760.5885 PC 34:1
(c)
CHCA (2step)+
LC-MS
down regulated
up regulated
MALDI-TOF MSI
-log10(p-value)
(d)
9-AA-
(e)
Selenocompound
Glutathione
D-Arginine and D-Ornithine
Beta-Alanine
Vitamin B6
Ubiquinone Biosynthesis
Thiamine
Riboflavin
Retinol
Pterine Biosynthesis
Porphyrin
Pantothenate and CoA Biosynthesis
One Carbon Pool by Folate
Nicotinate and Nicotinamide
Biotin
Sulfur
Oxidative Phosphorylation
Nitrogen
Caffein
Steroidgenesis
Androgen and Estrogen
Starch and Sucrose
Pyruvate
Propanoate
Phoshatidylinositol Phosphate
Glycolysis/Gluconeogenesis
Galactose
Fructose and Mannose
Citric Acid Cycle
Butyrate
Amino Sugar
Valine, Leucine and Isoleucine Degrad.
Tyrosine
Tryptophan
Phenylalanine and Tyrosine
Methionine
Lysine
Histdine
Glycine, Serine and Threonine
Arginine and Proline
Alanine, Aspartate and Glutamate
Steroid Biosynthesis
Primary Bile Acid Biosynthesis
Phospholipid Biosynthesis
Ketone Body
Glycerolipid
Fatty Acid
Fatty Acid Elong. in Mitochondria
Biosyntnthesis of Unsat. Fatty Acids
Arachidonic Acid
Alpha-Linolenic Acid
Pyrimidine
Purine
Bulk RNA-seq
9-AA-
9-AA+
CHCA (2step)-
CHCA (2step)+
NES
Amino Acid Metabolism
Biosynthesis of Secondary Metabolites
Carbohydrate Metabolism
Energy Metabolism
Lipid Metabolism
Endocrine System
Metabolism of Cofactors and Vitamins
Metabolism of Other Amino Acid
Nucleotide Metabolism

Supplementary Material Figure S6. Extended differential metabolite abundance between tumor and normal regions. (**a**) Percentage of total Wasserstein distance between tumor and normal regions explained by top 6 differentially abundant m/z peak bins for small molecule screen; (**b**) Percentage of total Wasserstein distance between tumor and normal regions explained by top 6 differentially abundant m/z peak bins for lipid screen; (**c**) Rank-rank hypergeometric overlap plots between LC-MS and MALDI-TOF MSI differential abundance results of small molecules for recrystallized CHCA matrix in positive polarity mode; smaller p-values indicate larger overlap between ranked lists; (**d**) Rank-rank hypergeometric overlap plots between LC-MS and MALDI-TOF MSI differential abundance results of small molecules for 9-AA matrix in negative polarity mode; smaller p-values indicate larger overlap between ranked lists; (**e**) Normalized enrichment score for KEGG pathways for each combination and bulk RNA-sequencing of tumor and normal regions for lipid screen.
